# Influence of Ag Nanoparticles with Different Sizes and Concentrations Embedded in a TiO_2_ Compact Layer on the Conversion Efficiency of Perovskite Solar Cells

**DOI:** 10.1186/s11671-018-2626-y

**Published:** 2018-07-13

**Authors:** Shuhan Li, Xiangyu Zhu, Bao Wang, Yu Qiao, Wenhui Liu, Hao Yang, Nan Liu, Mengwei Chen, Haifei Lu, Yingping Yang

**Affiliations:** School of Science, Wuhan University of Technology, Wuhan, 430070 China

**Keywords:** TiO_2_ compact film, Perovskite solar cells, Ag nanoparticles, Ag-embedded TiO_2_

## Abstract

In this study, Ag nanoparticles with diverse particle size and concentration, fabricated via the polyol method, were embedded in a TiO_2_ compact film to improve the power conversion efficiency of perovskite solar cells. Obtained results showed that Ag nanoparticles embedded in the TiO_2_ compact film do not affect the crystal structure of TiO_2_, while the size of the Ag nanoparticles can strongly influence the light absorption capacity of perovskite materials. However, the absorption intensity and power conversion efficiency of perovskite cells decreased with the increase in size of Ag nanoparticles. The amount of Ag nanoparticles was also an important factor for the performance of perovskite solar cells, and Ag nanoparticles in the compact layer were optimized to measure 10 nm in diameter, being embedded at a molar ratio of 1.5% (Ag:Ti = 1.5 mol%). Compared with hole-conductor-free perovskite solar cells that use carbon as counter electrodes, without Ag nanoparticles incorporated in the compact film, the enhanced efficiency of cells developed in this study can be mainly ascribed to the accelerated charge transfer, decreased charge recombination, and enhanced light absorption of the perovskite material in the visible region.

## Background

In recent years, with the gradual depletion of fossil energy, the search for sustainable new energy sources has become an important task. As a promising alternative, perovskite solar cells (PSCs) have attracted great interest because of their distinctive photovoltaic properties [1–5]. The power conversion efficiency (PCE) of PSCs has been significantly enhanced over time, from 3.8 to 22.1% [6–8]. Organo-metal halide perovskites are direct-bandgap materials with high carrier mobility, long charge diffusion, and large absorption coefficients [9–12]. These superior properties make them ideal photoactive materials in solar cells [4, 13–16].

The presence of a mesoporous layer determines the difference between mesoporous and planar structures. In general, the mesoporous structure is applied in high-efficiency PSC devices, as it increases the interface contact area to support film deposition and also improves charge extraction and charge transfer [17–19]. A typical PSC with mesoporous structure is made up of a fluorine-doped tin oxide (FTO) conductive layer, a compact layer, a mesoporous layer, a perovskite layer, a hole transport layer, and a counter electrode layer. In general, TiO_2_ has often been employed as the electron transport layer. However, other materials such as Al_2_O_3_, SnO_2_, and ZnO have also been used as photoanodes in PSC devices [20–25]. In fact, TiO_2_ nanoparticles play a dominant role in the transmission of electrons, which is why TiO_2_ is currently the preferred material for use in PSC devices. Under visible light irradiation, electron-hole pairs are generated in the perovskite layer of the PSC device, after which the electrons separately transfer to the electron transport layer (ETL), whereas the holes transfer to the hole transport layer [26]. The ETL includes two different layers, namely, the mesoporous layer and the compact layer. The compact layer is also known as the hole-blocking layer, as it can prohibit the recombination of electrons and holes when they meet on the surface of FTO conductive glass [1, 20, 27–29]. Therefore, high-quality compact films with surprisingly high carrier mobility characteristics and excellent electrical conductivity can have a significant impact on the efficiency of PSC devices. At the compact layer/mesoporous layer/perovskite layer interface, carrier recombination is reduced and electron injection can be accelerated. According to the research in recent years, to improve the PCE of a PSC device, the application of plasmonic nanoparticles has been proven feasible [29, 30]. Metal nanoparticles with surface plasmon resonance effect can increase the effective visible light absorption of the light absorption layer [29–31], which has been demonstrated through the application of metal plasmonic nanoparticles in different kinds of solar cells, like organic solar cells and silicon solar cells [32, 33]. Thus, the same method could be used to improve the PCE of PSC devices. Surface plasmons can be localized by noble metallic nanoparticles like Ag and Au. The excitation of localized surface plasmon resonance (LSPR) can be realized when the frequency of the incident visible light matches the resonance peak, which leads to unique optical properties, such as selective light extinction and enhancement of the electromagnetic field close to the surface of the metallic nanoparticles [34]. Therefore, the efficiency and photocurrent of a PSC device are improved after utilizing the optical properties of LSPR.

To the best of our knowledge, the effect of Ag nanoparticles (Ag NPs) embedded in TiO_2_ compact films on the efficiency of PSC devices has not been thoroughly investigated. Furthermore, over the past few years, many works have focused on plasmonic PSCs and organic photovoltaics [35–40], whereas Ag/TiO_2_ nanoparticle composites have been widely investigated during the past several decades for use in photocatalytic and dye-sensitized solar cells (DSSCs). Noble metal NPs have an impressive scattering effect and strong local surface plasmon resonance (LSPR). These characteristics can improve the photocurrent of DSSCs and enhance their photocatalytic capacity [41–48]. In our previous work, Ag-deposited TiO_2_ composites, TiO_2_ nanotube arrays, and rare-earth ion-doped nanomaterials were applied in DSSCs and PSCs [49–53]. In this work, a colloid was prepared with various sizes and concentrations of Ag NPs and embedded in a TiO_2_ compact layer to enhance the performance of PSCs. Results showed that the presence of Ag NPs in the compact film can increase the absorption of the PSC device under visible light irradiation. This leads to the formation of more photogenerated carriers due to the LSPR characteristic of Ag NPs compared to a similar device built without Ag NPs. Furthermore, the optimized size and concentration of Ag NPs in the TiO_2_ precursor are about 10 nm and 1.5 mol%, respectively, which can induce the highest power conversion efficiency of the PSC device.

## Methods

Various approaches have been developed for the preparation of size-controlled Ag NPs [38, 54–56]. In this study, we chose the chemical method of fabricating Ag NPs of different sizes because of the easily available chemical materials and controllable protocol. For Ag NPs of 10 nm in diameter, 0.75 g polyvinylpyrrolidone (PVP, K30) were dissolved in 50 ml ethylene glycol. After the PVP solution was heated up to 120 °C, 0.25 g silver nitrate (AgNO_3_) dissolved in 25 ml ethylene glycol was added dropwise and allowed to react for 1 h at this temperature. A light brown colloidal solution was formed, which implied the formation of Ag NPs. For Ag NPs of 30 nm in size, 1.5 g PVP were added to 20 ml ethylene glycol to be fully dissolved and heated up to 120 °C, then 0.25 g AgNO_3_ dissolved in 10 ml ethylene glycol was added dropwise into the heated solution and allowed to react for 1 h at this temperature. The color of the solution was brown after the 1-h reaction. For Ag NPs of 40 nm in size, 1.5 g PVP were added to 20 ml ethylene glycol to be fully dissolved and heated up to 120 °C, then 0.15 g AgNO_3_ dissolved in 10 ml ethylene glycol was added dropwise into the heated solution and allowed to react for 1 h at this temperature. The color of the solution was brown after the 1-h reaction. For Ag NPs of 55 nm, the basic procedure and the amount of raw material were the same as used for the 30-nm Ag NPs, but the heating temperature was 150 °C in order to form larger Ag NPs. The solution gradually turned dark brown after a 30-min reaction. After cooling to room temperature, all the solutions were washed in order with deionized water, ethanol, and acetone, followed by drying at 50 °C overnight in a vacuum drying oven. Hence, Ag NPs of four different sizes were obtained.

The TiO_2_ compact precursor solution was prepared by adding 1 ml titanium diisopropoxide bis (75%, Sigma-Aldrich, USA) to 19 ml ethanol. In order to prepare precursors containing Ag NPs of different sizes and concentrations, different amounts of Ag NPs were added in the TiO_2_ precursor to obtain various molar ratios of Ag to Ti, i.e., 0.5, 1, 1.5, 2, and 2.5 mol%, and stirred for 1 h at room temperature to form uniform precursor solutions. For the precursor of the mesoporous layer, ZrO_2_ or TiO_2_ colloidal solution was added to ethanol, at a mass ratio of 1:5, and stirred for 12 h at room temperature.

The glass/FTO substrates were pre-cleaned by deionized water (with detergent), acetone, isopropanol, and ethanol, in sequence, in an ultrasonic box. For the fabrication of the PSC devices, TiO_2_ sol-gel precursors with different sizes of Ag NPs were firstly spin-coated on the transparent electrode at 4000 rpm for 20 s, then heated at 150 °C for about 10 min. The above procedures were repeated for the fabrication of the compact film, which was finally formed after annealing at 500 °C for 30 min. The same method was used to fabricate the pristine TiO_2_ compact film without any metal NPs. Then, the substrates with compact film were immersed into an aqueous solution of TiCl_4_ for 30 min, at 70 °C, and subsequently heated at 150 °C for approximately 10 min to optimize the TiO_2_ compact layer.

The mesoporous TiO_2_ layer was deposited on top of the compact film via spin-coating of the TiO_2_ colloidal solution at 3500 rpm for 20 s, followed by heating at 150 °C for 10 min and annealing at 500 °C for 30 min to create the anatase TiO_2_ mesoporous layer. It has been shown that the adoption of ZrO_2_ in PSCs can enhance their stability [57]. Herein, we additionally used a ZrO_2_ colloidal solution for spin-coating on the anatase TiO_2_ mesoporous film, and the obtained ZrO_2_ film was sintered at 500 °C for 30 min. After the substrate was cooled to room temperature, the FA_0.4_MA_0.6_PbI_3_ perovskite layer was formed by spin-coating the precursor solution at 1000 rpm for 10 s and 4000 rpm for 30 s. The perovskite precursor solution (FA_0.4_MA_0.6_PbI_3_) contained 462 mg PbI_2_, 95.4 mg methylammonium iodide (CH_3_NH_3_I, 99.99%), and 68.8 mg formamidinium iodide (HN=CHNH_3_I, 99.99%), which were dissolved in 600 mg *N*,*N*-dimethylformamide and 78 mg dimethyl sulfoxide. During the spin-coating of the perovskite precursor, 1 ml diethyl ether was added evenly to form a stable perovskite film, according to a previous report [58], which was then heated at 100 °C for 10 min. The PSC device was obtained after the carbon counter electrodes (30 μm) were built by screen-printing and then annealing at 100 °C for 30 min.

A field emission scanning electron microscope (Zeiss Ultra Plus, Germany) and transmission electron microscope (TEM; JEM-2100F, Japan) were used to investigate cross-sections of the PSC devices, as well as the surface morphology of the Ag NPs and compact film. The crystal structure of Ag and TiO_2_ nanoparticles was obtained by an X-ray diffractometer (XRD; MAX-RB RU-200B, Japan). The surface of the compact layer samples was measured by X-ray photoelectron spectroscopy (XPS; ESCALAB 250Xi, Thermo Fisher Scientific). Current density-voltage (*J*-*V*) curves of the PSCs were tested by a solar light simulator (Oriel Sol3A, Newport Corporation, USA), under AM 1.5G illumination, at 100 mW/cm^2^ intensity. The absorption spectra were determined by ultraviolet-visible spectroscopy (UV-vis; Shimadzu, Japan). Incident photon-to-electron conversion efficiency (IPCE; Newport Corporation, USA) was used to investigate the quantum efficiency of the PSC devices.

## Results and Discussion

For spherical NPs, if their size is much smaller than the wavelength of the incident light, the quasi-static approximation can be used to describe their LSPR properties. According to the Mie theory, and by applying quasi-static approximation, scattering plays a leading role with the increasing radius of the spherical NPs, and the extinction intensity is then mainly determined by the scattering. Additionally, the absorption gradually affects the extinction intensity when the radius of the spherical NPs is decreasing [59]. However, the extinction intensity is also related to the charge trap state of the spherical NPs and the dielectric constant of the surrounding medium. Thus, the extinction intensity needs further study.

**Fig. 1:**
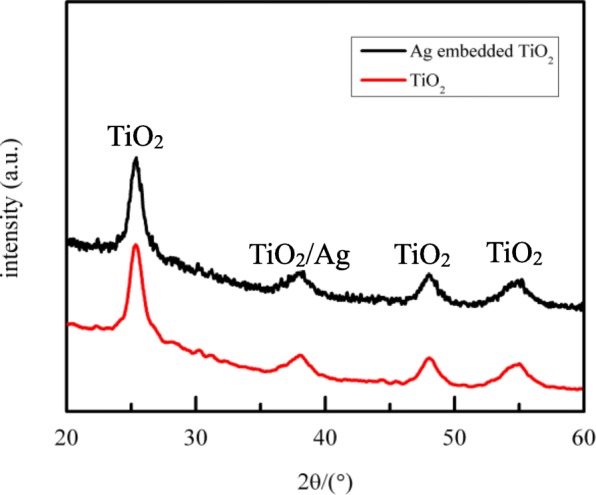
XRD spectra of TiO_2_ and Ag-embedded TiO_2_

Figure 1 shows the XRD patterns of the TiO_2_ and the Ag-containing TiO_2_. It is obvious that all XRD curves have strong peaks attributed to anatase TiO_2_ with the standard anatase PDF card, which indicates that the crystal structure of TiO_2_ hardly changes with the addition of Ag. In fact, the peaks of Ag cannot be observed directly because of the low concentration of Ag and the coinciding peak at about 2*θ* = 38°.

**Fig. 2:**
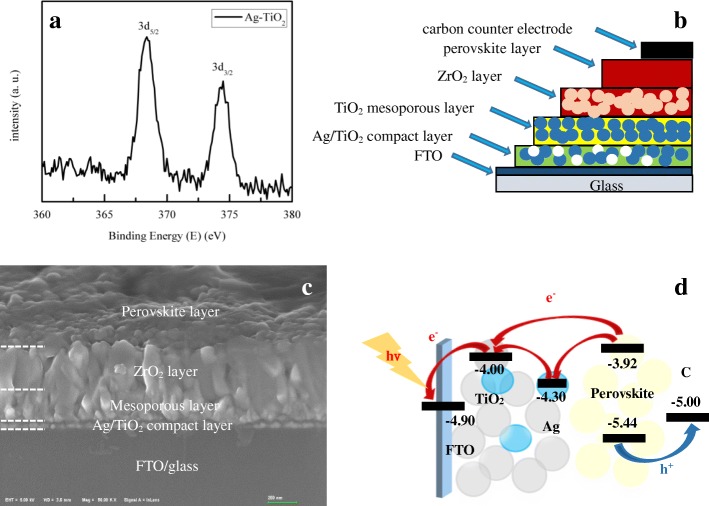
**a** Ag 3d XPS spectra of Ag/TiO_2_ composites. **b** Device structure of a Ag/TiO_2_ perovskite solar cell. **c** A cross-sectional FE-SEM image of Ag/TiO_2_ perovskite solar cell. **d** Schematic diagram of the photo generated carriers separation and transfer (the gray, blue, and yellow circles represent anatase TiO_2_, Ag, and perovskite nanoparticles)

To investigate the chemical elements of the TiO_2_ composite compact film, XPS was used to explore the chemical binding energy of the Ag NPs embedded in the TiO_2_ compound in the compact film. Figure 2a shows the electron-binding energy of Ag 3d with the composite compact film. The peaks of Ag 3d_5/2_ and Ag 3d_3/2_ were located in 368.3 and 374.3 eV, which is consistent with the standard binding energy of Ag^0^ [42, 43]. This implies that the Ag elements in the compact film were present in the form of simple substances, without any chemical reaction. Figure 2b, c shows the device structure and the cross-sectional image of the PSC device with integrated Ag NPs in the compact layer. In fact, the size of ZrO_2_ and TiO_2_ is almost the same of about 20 nm. Thus, the interface between ZrO_2_ and TiO_2_ is not easy to identify.

**Fig. 3:**
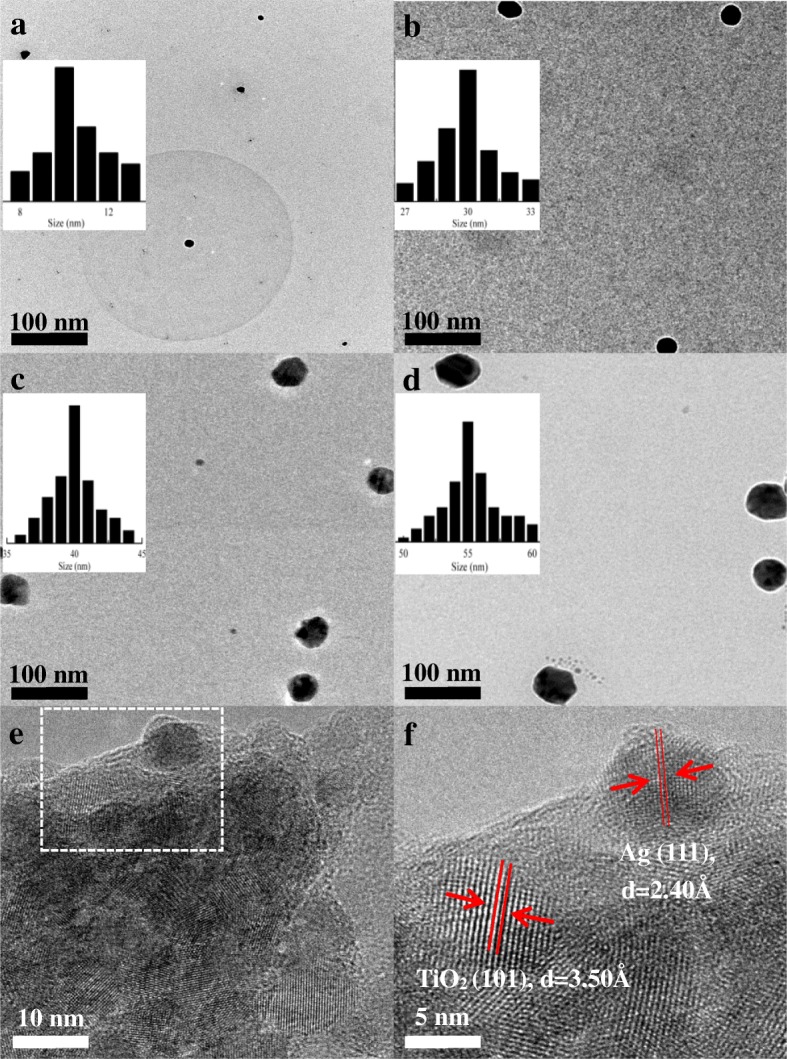
HRTEM images and size distribution results of **a** 10-nm Ag NPs, **b** 30-nm Ag NPs, **c** 40-nm Ag NPs, and **d** 55-nm Ag NPs; HRTEM images of **e** Ag/TiO_2_ composite compact film and **f** enlarged image from the dotted area of **e**

Since the distribution of Ag cannot be precisely distinguished through SEM imaging, TEM images were used to assess the size of both the Ag NPs and the Ag/TiO_2_ composite compact film. TEM images showing different sizes of Ag NPs and Ag/TiO_2_ composites are presented in Fig. 3. Figure 3a–d shows the different structures and size distribution results of Ag NPs with the sizes of 10 nm (Fig. 3a), 30 nm (Fig. 3b), 40 nm (Fig. 3c), and 55 nm (Fig. 3d), which were prepared by the polyol method. As observed in the abovementioned figures, the sizes of individual Ag NPs showed little variation and the uniform size of the Ag NPs could be easily distinguished. Figure 3f shows an enlarged image of Ag/TiO_2_ composites in the compact film from the dotted area in Fig. 3e. The Ag NPs were encircled by TiO_2_ for the formation of Ag/TiO_2_ composites, and the lattice fringes of Ag and TiO_2_ NPs were about 2.40 and 3.50 Å from the dotted area in Fig. 3f.

Figure 2e represents the schematic diagram of the photogenerated carrier separation and transfer in the Ag/TiO_2_ PSC device under visible light irradiation, without the ZrO_2_ layer (red arrows indicate the electron transport pathway, and the blue arrow represents the hole transport pathway). For the TiO_2_ compact layer containing the Ag NPs, the Ag NPs can act as charge trap sites, which is because the conduction band of Ag NPs is between the TiO_2_ and the perovskite material [35]. In general, the LSPR of Ag can improve the absorbance of both organic and perovskite solar cells significantly [35, 36, 44, 45]. Thus, the management of absorbance and charge trapping through using Ag NPs of different sizes and concentrations should influence the strength of the photocurrent and the performance of the PSC device.

**Fig. 4:**
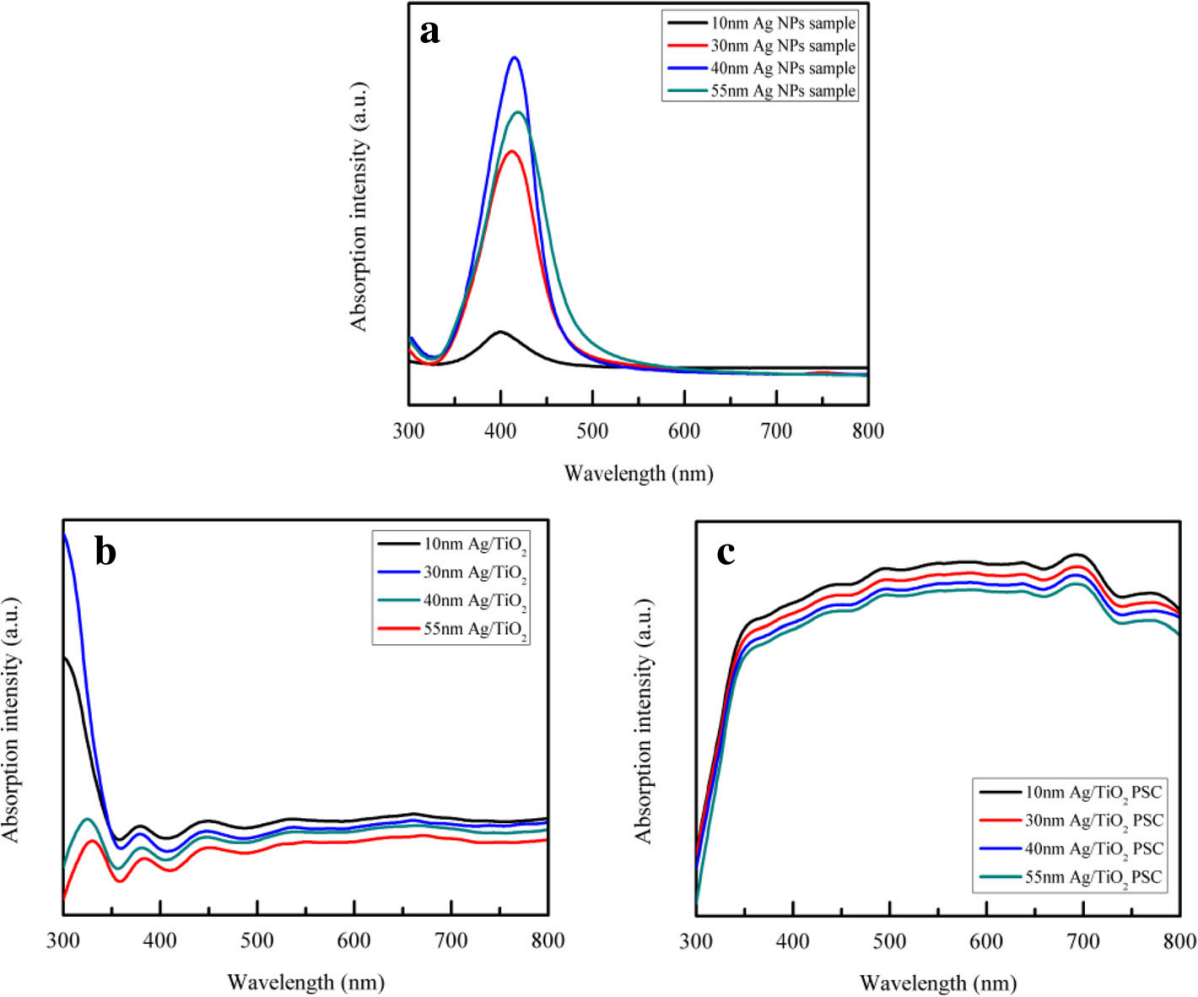
UV-vis absorption spectra of the **a** different sizes of Ag NP sample, **b** Ag/TiO_2_ samples with various sizes of Ag NPs, and **c** Ag/TiO_2_ PSC device samples with various sizes of Ag NPs

**Fig. 5:**
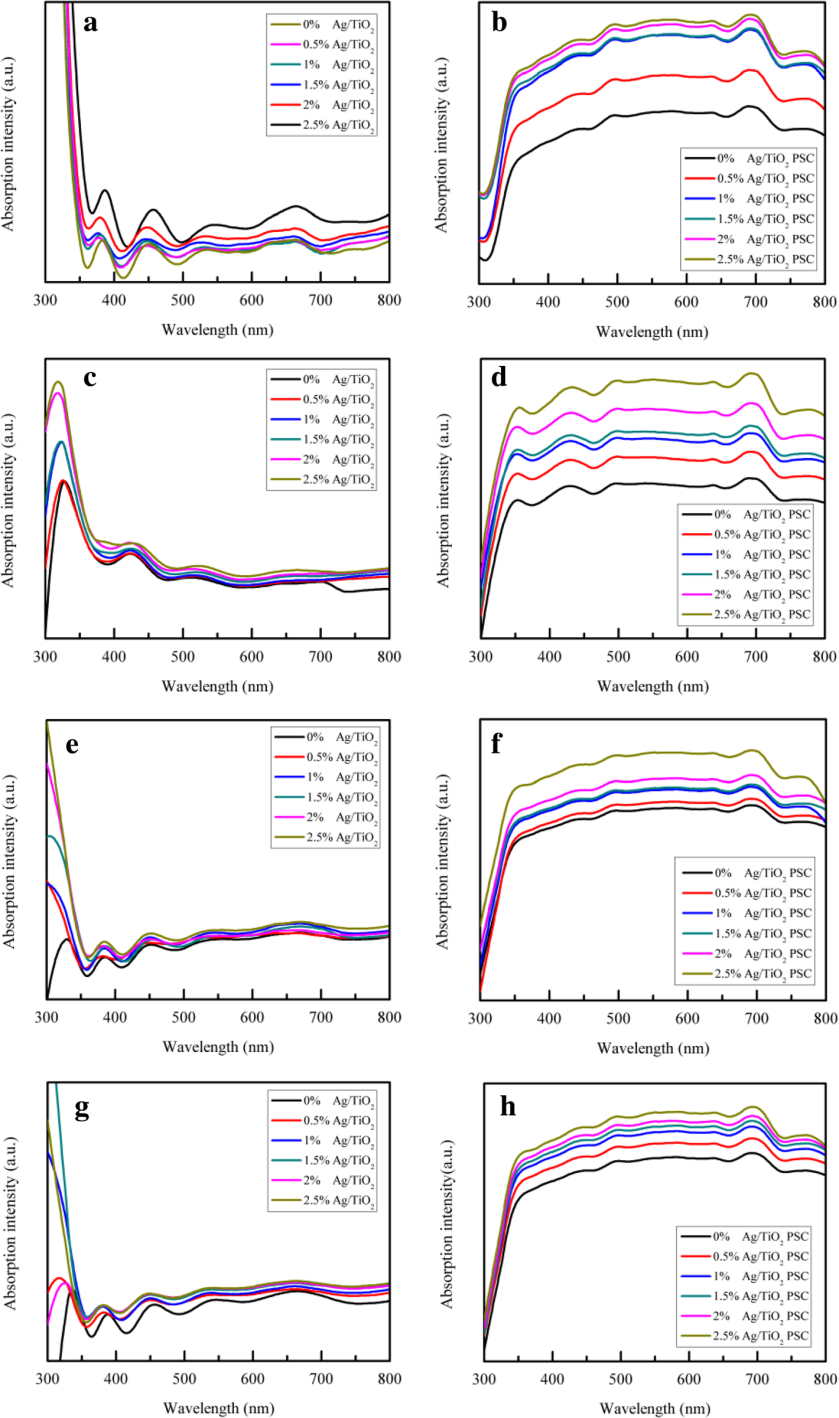
UV-vis absorption spectra of the compact films and the whole PSC devices with **a**, **b** 10-nm Ag NPs; **c**, **d** 30-nm Ag NPs; **e**, **f** 40-nm Ag NPs; and **g**, **h** 55-nm Ag NPs

Figure 4a presents the absorbance spectra of Ag NPs of different sizes in water, which is indicated by the different absorption peaks. The corresponding absorption peaks of 10-, 30-, 40-, and 55-nm Ag NPs were approximately 400, 410, 415, and 420 nm, respectively. In addition, the UV-vis absorption spectra of the Ag/TiO_2_ composite compact films and the whole PSC devices containing Ag NPs of various sizes and different concentrations are respectively shown in Figs. 4 and 5. As seen in Fig. 4b, the absorbance of 10-nm Ag NPs in the Ag/TiO_2_ composite compact film was higher than that of the other sizes, and the absorbance varied inversely with the size of the Ag NPs. In fact, the absorption spectra in Fig. 4c follow the same trend as depicted above in Fig. 4b, decreasing with the increase in Ag NP size. This may be due to the increased optical loss caused by the reflection of larger Ag NPs and the intrinsically strong LSPR effect of small-sized Ag NPs. According to previous research, as the size of noble mental NPs increases, the LSPR effect gradually decreases and light scattering becomes more dominant [37, 46, 47]. Thus, the LSPR effect has a great impact on the absorbance of PSC devices when the Ag NPs are relatively small.

Figure 5a–h shows the UV-vis absorption spectra of the compact films and the whole PSC devices with 10-, 30-, 40-, and 55-nm Ag NPs. As observed, with the increase in Ag content, the absorption of the compact film was gradually enhanced in the completely visible region. The absorption of the whole PSC device followed the same trend as the content of Ag increased. These spectra collectively indicate that 2.5 mol% content of Ag/TiO_2_ had the highest absorption at every unique Ag NP size, which is because of the LSPR effect. With the increase in content of Ag NPs, the LSPR effect also became stronger. Nanoparticle size plays an important role in the extinction behavior, such as the light absorption and scattering. Comparing Fig. 4 with Fig. 5a, c, e, g, the plasmonic absorption peaks of 10-, 30-, 40-, and 55-nm Ag NPs shifted when the Ag NPs were embedded in the TiO_2_ compact layer, which is ascribed to the larger refractive index of TiO_2_ compared to water [48]. In fact, TiO_2_ NPs have a relatively high absorption in this wavelength region. Although the LSPR effect of the Ag NPs occurs whenever under light irradiation, the absorption at a wavelength region shorter than 350 nm does not show a significant increase because of the high extinction coefficient of the perovskite material which leads to the saturation of light absorption. The absorbance of the perovskite material is strong in the short wavelength region of about 400 nm and relatively weak in the long wavelength region of 600~800 nm. Due to the LSPR effect of the Ag NPs, the absorbance of the whole PSC device is significantly enhanced. Moreover, the Ag NPs enhance light absorption in the visible region (380~780 nm), which can be mainly attributed to the LSPR of Ag NPs, in addition to the absorption of the perovskite material, when the size of the Ag NPs is smaller than ~ 100 nm [46].

**Fig. 6:**
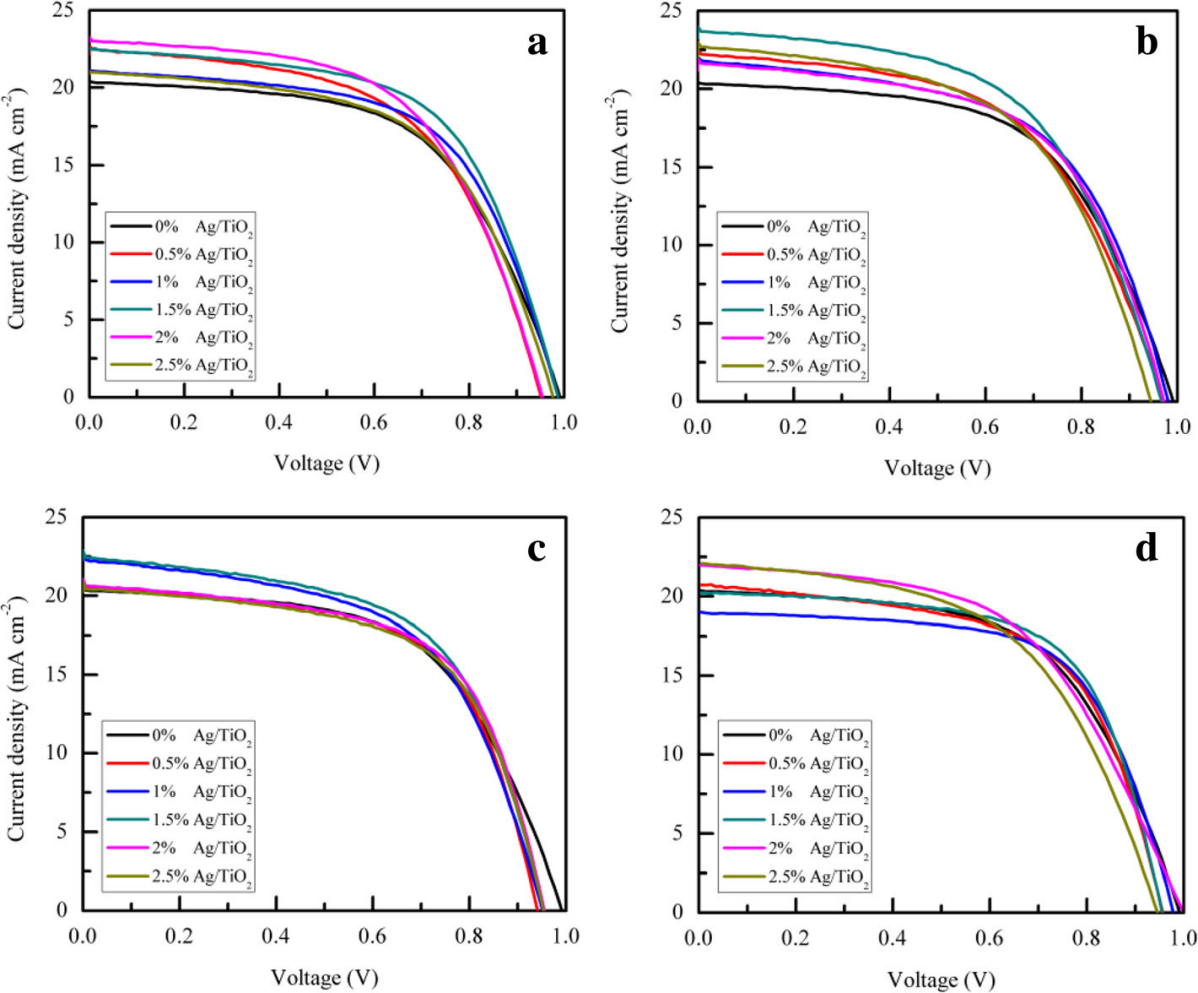
*J*-*V* curves of PSC devices with different content of various size of Ag NPs of **a** 10 nm, **b** 30 nm, **c** 40 nm, and **d** 55 nm

**Fig. 7:**
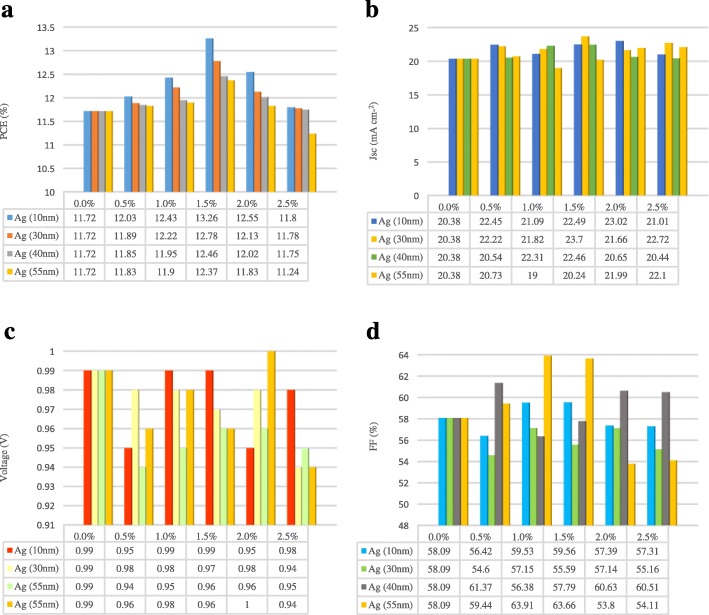
Two-dimensional histograms and the corresponding tables of **a** PCE, **b** *J*_sc_, **c** voltage, and **d** FF of PSC devices with different sizes and content of Ag NPs

The measured *J*-*V* curves revealing the performance of the PSC devices with different sizes and content of Ag NPs are shown in Fig. 6. Figure 7 represents a two-dimensional diagram and the corresponding table of PCE, short-circuit current density (*J*_sc_), open-circuit voltage (*V*_oc_), and fill factor (FF) of the Ag/TiO_2_ PSC devices with different content and sizes of Ag NPs. In fact, compared with the devices without Ag NPs, the devices with different sizes of Ag NPs and different Ag content showed little difference in *V*_oc_. For the Ag NP size of 10, 30, 40, and 55 nm, the *J*_sc_ of the Ag/TiO_2_ PSC devices were all higher than those of the devices without Ag NPs (20.38 mA cm^−2^), the corresponding values being approximately 23.02, 23.7, 22.46, and 22.1 mA cm^−2^, respectively. The improved *J*_sc_ of the Ag/TiO_2_ PSC devices can be ascribed to the perovskite material absorption enhancement by LSPR. Thus, the incorporation of Ag NPs can improve the PCE of PSC devices by enhancing the absorption intensity of the perovskite material. For the same content of Ag NPs, with the increase in size of Ag NPs, the PCE gradually decreases. The increased optical loss caused by the reflection and absorption of larger Ag NPs decreases the light absorption of the perovskite material. Based on the table in Fig. 7, the PCE of the Ag/TiO_2_ PSC devices varies inversely with the size of the Ag NPs, and 1.5 mol% Ag/TiO_2_ PSC devices with 10-nm Ag NPs had an average PCE of 12.01% (out of 10 PSC devices) and the highest PCE of 13.26%. For the Ag NP size of 10 nm, the *J*_sc_ of the 1.5 mol% Ag/TiO_2_ PSC devices were slightly lower than those of the 2 mol% Ag/TiO_2_ PSC devices, but the 1.5 mol% Ag/TiO_2_ PSC devices had the highest FF when compared to all other content of 10-nm Ag/TiO_2_ PSC devices. However, with the increase in Ag NP content, the PCE of Ag/TiO_2_ PSC devices with different sizes of Ag NPs decreased gradually, which is because of the charge trapping sites of the Ag NPs, which decrease the performance of the PSC. In fact, Ag NPs are considered to trap the electrons generated by perovskite materials and can impede the transport of charges because of the different energy levels between TiO_2_ and the Ag NPs [60]. Thus, the combined effect of light scattering and LSPR can significantly influence the performance of Ag/TiO_2_ PSC devices.

**Fig. 8:**
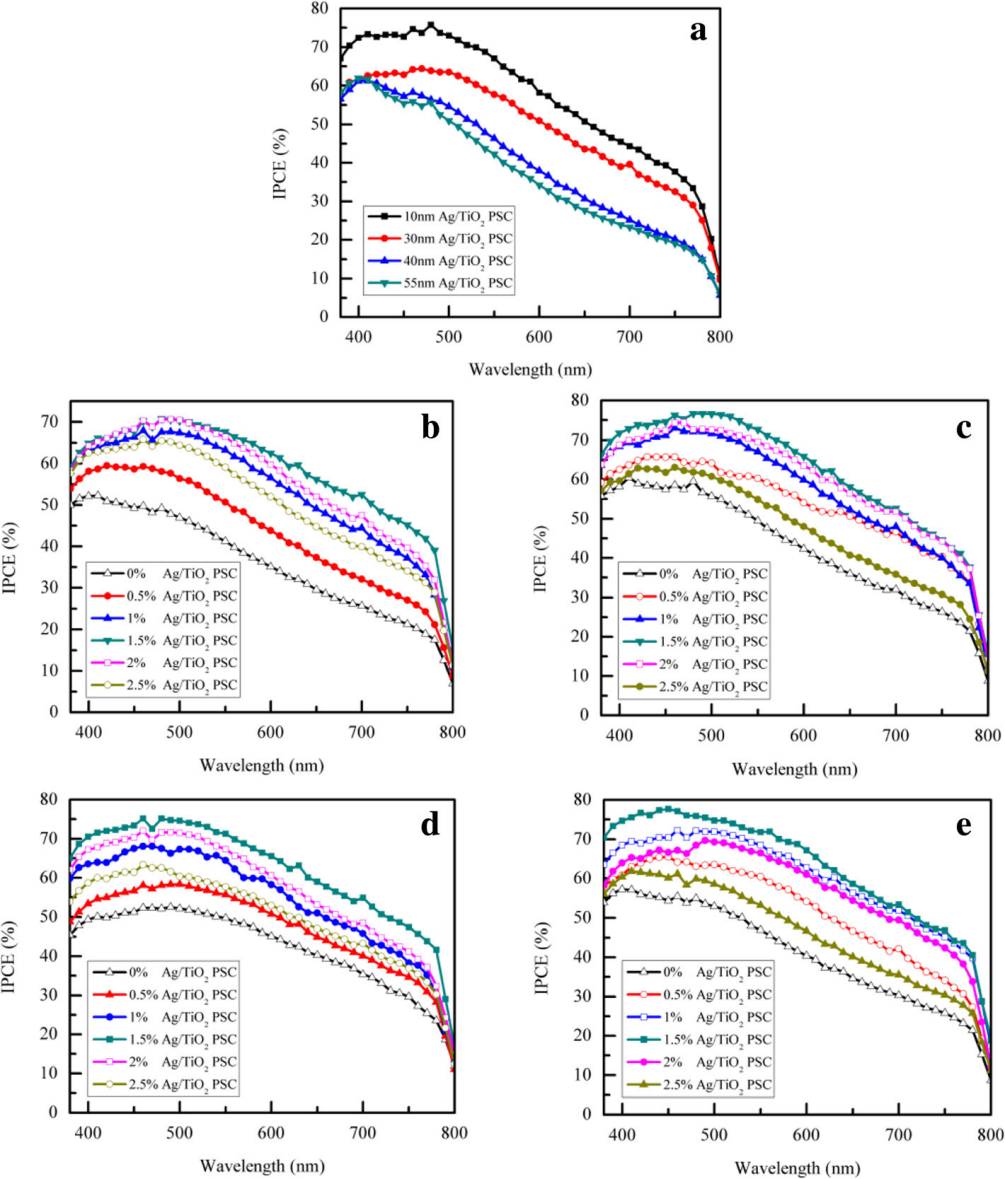
IPCE spectra (380 to 800 nm) of PSC devices with **a** different sizes of Ag NPs, **b** different content of 10-nm Ag NPs, **c** different content of 30-nm Ag NPs, **d** different content of 40-nm Ag NPs, and **e** different content of 55-nm Ag NPs

Figure 8 represents the IPCE curves of the PSC devices with or without Ag NPs. The PSC device with the 10-nm Ag NPs showed the highest enhancement of the IPCE in the visible region, as depicted in Fig. 8a. Moreover, low enhancement values can be observed when the size of the Ag NPs was bigger than 10 nm. In general, the PSC devices with Ag NPs had higher IPCE enhancement values compared to samples without Ag NPs, which is mainly due to the absorption enhancement by the LSPR of Ag NPs. It can be observed in Fig. 8b–e that the IPCE enhancement values of 1.5 mol% Ag/TiO_2_ PSC devices were highest for every Ag NP size. In addition, the IPCE enhancement values of the PSC devices decreased gradually with the increase in Ag content, which may ascribe to the charge trapping on the Ag NPs and deterioration of the electron transport lowering the PCE. Finally, the IPCE is decreased by the electron transfer to the Ag NPs, where the charges are trapped by the barriers between TiO_2_ and the Ag NPs.

## Conclusions

In this article, we used the polyol method to prepare Ag NPs of different sizes and investigated the influence of size and content of Ag NPs on PSC devices. The absorption enhancement of the PSC devices in the visible region was mainly ascribed to the LSPR of Ag NPs. With the increase in size and content of Ag NPs, the absorption intensity of both TiO_2_ compact film and PSC device gradually decreased and increased, respectively. With the addition of Ag NPs, the charge transport capability increases and the performance of the PSC device is consequently improved. Moreover, the PSC device with a small size and amount of Ag NPs showed higher PCE and IPCE values. Specifically, the PSC device with 10-nm Ag NPs and 1.5 mol% Ag/TiO_2_ compact film exhibited the highest PCE of 13.26%. These results can provide a reference for introducing different sizes and content of Ag NPs into PSC devices in order to improve their performance.
